# The Roles of Hedgehog Signaling in Upper Lip Formation

**DOI:** 10.1155/2015/901041

**Published:** 2015-09-03

**Authors:** Hiroshi Kurosaka

**Affiliations:** Department of Orthodontics and Dentofacial Orthopedics, Graduate School of Dentistry, Osaka University, Osaka 565-0871, Japan

## Abstract

Craniofacial development consists of a highly complex sequence of the orchestrated growth and fusion of facial processes. It is also known that craniofacial abnormalities can be detected in 1/3 of all patients with congenital diseases. Within the various craniofacial abnormalities, orofacial clefting is one of the most common phenotypic outcomes associated with retarded facial growth or fusion. Cleft lip is one of the representative and frequently encountered conditions in the spectrum of orofacial clefting. Despite various mechanisms or signaling pathways that have been proposed to be the cause of cleft lip, a detailed mechanism that bridges individual signaling pathways to the cleft lip is still elusive. *Shh* signaling is indispensable for normal embryonic development, and disruption can result in a wide spectrum of craniofacial disorders, including cleft lip. This review focuses on the current knowledge about the mechanisms of facial development and the etiology of cleft lip that are related to *Shh* signaling.

## 1. Introduction

The proper growth and fusion of embryonic facial processes are critical for craniofacial development, and failure of either step can lead to a wide variety of orofacial clefting. Cleft lip and/or palate (CL/P) is one of the most common orofacial clefts and is found in 1/700 living newborns [1]. CL/P has a lot of variation in terms of the degree of the cleft, such as cleft lip (CL), cleft of the secondary palate (CP), and CL/P [2, 3]. Most of the time, all of these cleft phenotypes are considered to be the same disorder with different severity, since the facial processes share a similar cellular context (mesenchymal cells surrounded by facial ectoderm). However, from the anatomical point of view, the lip and secondary palate have different origins, with the lip being formed by the fusion of medial and lateral nasal processes, while the secondary palate is a structure of fused palatal shelves that originate from maxillary processes. In addition, the timing of fusion is different between these two structures. For these reasons, it is worthwhile to consider the different cleft etiologies separately.

Many signaling pathways have been revealed to be associated with the etiology of CL/P [4]. Sonic hedgehog (*Shh*) signaling is one of the most important signaling pathways for the development of many organs, including craniofacial structures [5], and either a loss of function or gain of function of this signaling pathway can lead to craniofacial abnormalities, including CL/P [6, 7]. The mechanism of* Shh* signaling in secondary palate development has been well studied [8–11]; however, there have been a limited number of studies focused on lip development. In this review, we focus on and discuss the roles of* Shh *signaling during lip formation by summarizing the current knowledge based on many animal model studies including mice (Table 1).

## 2. The Development of Facial Processes Required for Lip Formation

**Figure 1:**
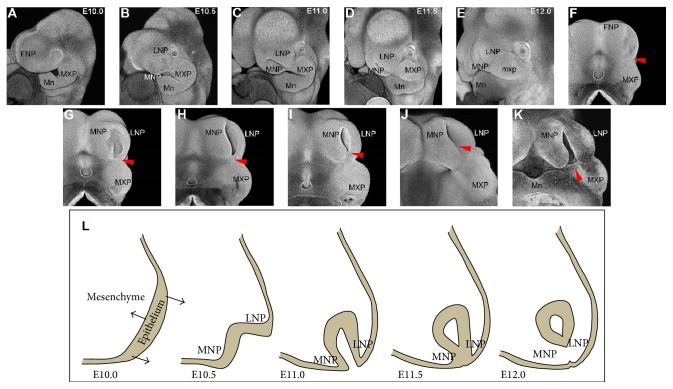
The growth of facial processes in mouse embryos shown by whole mount nuclear fluorescent imaging. ((A)–(E)) Oblique views of the facial development of mouse embryos from E10 to E12.0. ((F)–(J)) The same heads were captured from the ventral side of the head with the mandible removed. (K) The representative phenotype of cleft lip, which was induced by whole embryo culture. The red arrowheads show the position where the facial processes fuse. (L) Cartoon sequence of growing nasal processes at each stage. Black arrow shows the direction of processes growth and folding. MNP: medial nasal process, LNP: lateral nasal process, MXP: maxillary process, and Mn; mandible.

In mice, the development of the medial nasal process (MNP) and lateral nasal process (LNP), which are the processes which eventually give rise to the nose and part of the lip, are not visibly evident on embryonic day (E)10.0, and the frontonasal process (FNP) still has a relatively flat structure (Figures 1(a) and 1(f)). After around E10.5, the MNP and LNP start to grow out from the FNP (Figures 1(b) and 1(g)). These processes continue to grow and begin to fuse at the position called the lambdoidal region (Figures 1(c)–1(e) and 1(h)–1(j), red arrowhead) where MNP, LNP, and maxillary process (MXP) integrate together (Figures 1(c)–1(e) and 1(h)–1(j)). The growth and fusion of these developing processes are crucial for normal midface development, including lip formation. If these processes fail to fuse, it can lead to CL (Figure 1(k)). Several studies have shown that the surgical removal or mechanical inhibition of facial processes results in cleft lip in rat embryos [12, 13]. Interestingly, the growth and fusion of the facial processes at these stages seem to be largely evolutionally conserved among many species, which makes animal models useful for investigating the mechanisms underlying human cleft lip [14–16].

## 3. The Role of* Shh* Signaling in Removing Epithelial Seam Cells

At the lambdoidal region, the epithelial seam cells between fusing processes have to break down in order to form a mesenchymal bridge (Figure 1(l)). A few mechanisms have been proposed to explain this breakdown of the epithelial seam, such as apoptosis or the epithelial mesenchymal transition (EMT). Similar to fusion of the secondary palate, active cell death can be observed in the epithelial seam between the fusing MNP and LNP in mice [7, 14]. However, mice that lacked this apoptosis due to inhibition of the caspase signaling pathway did not show CL but did have secondary palate malformation and exencephaly [17, 18]. These results suggested that there are other mechanisms leading to the removal of the epithelial seam cells. Some studies have shown that the EMT occurs in epithelial seam cells in chick fusing facial processes [19]. However, there have been no experiments that have inhibited the EMT completely from these fusing processes, so the mechanism by which the epithelial seam cells are removed is not fully understood.

Another epithelial cell that plays a critical role for fusing facial processes is the periderm cells, which slough off from the growing processes to allow for proper fusion [20]. In the mouse secondary palate, synergistic mutations of* P63* and* Irf6* lead to excessive layers of periderm cells, which results in cleft palate [21, 22]. On the other hand,* Irf6* null mice showed a lack of periderm cells, which was shown to be associated with ectopic fusion of the secondary palatal shelf to the tongue [23]. Additionally,* Irf6* was shown to allow the epithelial cells to exit the cell cycle in order to remove the epithelium [21]. In our previous study, we discovered that a disturbed* Shh* signaling gradient resulted in perturbed* P63* and* Irf6* expression, which possibly led to persistent SSEA1-positive periderm cells on the MNP, resulting in the CL phenotype, together with alterations in the balance of cell proliferation and apoptosis at the epithelial seam, which caused a failure in the removal of the epithelial seam [7]. However, the relationship between* Shh* signaling and periderm cell development and the relationship between periderm cell defects and the etiology of CL are still elusive and require further studies.

## 4. The Expression and Role of* Shh* Signaling during the Growth and Fusion of Facial Processes

**Figure 2:**
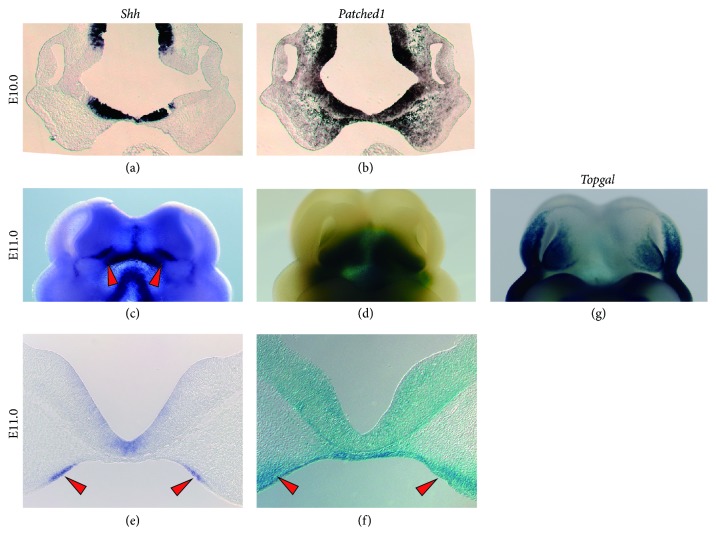
The expression of* Shh* and* Patched1* during the growth of facial processes in mouse embryos. ((a), (c), and (e))* In situ* hybridization of* Shh* on E10 and E11.0. (b)* In situ* hybridization of* Patched1* on E10. ((d) and (f))* LacZ* staining of E11.0* Patched1-LacZ* mice. (g)* LacZ* staining of a* Topgal* mouse embryo on E11.0. The red arrowheads indicate the position of the frontonasal ectodermal zone (FEZ).

*Shh* signaling plays various important roles in craniofacial development. In addition, the expression of* Shh* shows a unique pattern during the outgrowth and fusion of nasal processes. Before evident outgrowth of the MNP and LNP,* Shh* is expressed at the ventral neural tube, but not at the nasal process (Figure 2(a)). At the same stage, mesenchymal cells in the frontonasal processes are already receiving the SHH ligand to activate the* Hedgehog* (Hh) signaling pathway, as shown by* Patched1* expression, which is one of the receptors and markers of* Hh* signaling (Figure 2(b), asterisk).* Patched1* expression becomes stronger at the center of the face, which may be caused by the high amount of SHH ligand secreted from the ventral neural tube. Around E11.0 in mice,* Shh* expression started to be observed at specific regions of the oral ectoderm, followed by expanded* Patched1* expression, mainly in the MNP (Figures 2(c)–2(f)). It is well known that this intraoral* Shh-*expressing region, together with the* Fgf8* expressing domain, plays a critical role in the growth and patterning of the FNP, which is called the frontonasal ectodermal zone (FEZ) [24, 25]. The* Shh* expression at the FEZ region is known to require* Shh* signaling from the ventral neural tube and* Bmp* signaling from the FNP [26, 27]. Genetic ablation of* Shh* during mouse development tended to lead to a smaller MNP or diminished MNP development, which was associated with severe cell death and holoprosencephaly, while the LNP developed relatively normally, as shown by analyzing molecular markers such as* Pax9* or* Pax7* [28–30]. Synergistic mutations in* Boc* and* Gas1,* which are receptors of SHH ligand, led to CL with holoprosencephaly [31]. In addition, the prenatal administration of a* Shh* signaling inhibitor could cause a similar phenotype, with wide variation [6, 32].

On the other hand, enhanced* Shh* signaling due to mutated* Patched1* during head development could also result in CL with severe craniofacial abnormalities, including hypertelorism [7]. These results, in conjunction with the induction of CL by surgical removal of facial prominences in rat embryos [12], indicate that adequate MNP development, governed by proper* Shh* signaling from the ventral neural tube and consequently at the FEZ region, is essential for the fusion of facial processes and lip formation. It is also important to understand the tissue-specific roles of* Shh* signaling during lip fusion. Mutant mice that had* Patched1 *conditionally knocked out from cranial neural crest cells also showed a CL phenotype [33].

*Smoothened (Smo)* is another critical mediator of* Shh* signaling [34]. A previous study showed that inhibiting the activity of* Smo* in cranial neural crest cells was associated with craniofacial abnormalities, such as a dramatically truncated face, which included a severely deformed lip, together with hypoplastic cranial bones [35]. Conversely, the overexpression of* Shh* in the facial ectoderm was also associated with lip abnormalities with CP [36]. These results indicate that adequate* Shh* signaling in both the developing facial ectoderm and neural crest cells is essential for proper lip formation. Subsequently,* Shh* also starts to be expressed at the ventral nasal pit epithelium while the MNP and LNP are fusing [35, 37, 38]. The role of this* Shh* signaling in lip formation is still unclear and requires further investigation by removing or enhancing* Shh* signaling in a tissue- and stage-specific manner.

## 5. Cilia-Associated* Shh* Signaling and Midfacial Development

Primary cilia are thin cellular processes that extend from the surface of various types of cells. It is well known that cilia have important roles in a variety of signaling pathways which are indispensable for proper development or metabolism [39]. Primary cilia are known to work as mediators of* Shh* signaling by analyzing the localization of PATCHED1 and SMOOTHENED, which are the important downstream factors required for activating* Shh* signaling in response to the ligand [34]. In humans, there is a disease spectrum of ciliopathy caused by mutations of the genes important for ciliogenesis, such as intraflagellar transport proteins (IFTs). These exhibit a wide spectrum of phenotypes, including craniofacial defects [39, 40]. Previous reports showed multiple mice mutants that had disrupted ciliary proteins that were associated with craniofacial abnormalities.

Interestingly, many of these mutants had phenotypes that could have resulted from disrupting* Shh* signaling, such as polydactyly or anencephaly. Neural crest-specific elimination of* Kif3a*, one of the intraflagellar transport proteins in mice, caused excessive* Shh* signaling with hypertelorism and a midfacial cleft [41]. Mutation of* Ift172* in mice leads to CP and recapitulates the phenotype of human VACTERL [42, 43]. Disrupting* Ift144* led to CL with anencephaly and polydactyly, which are representative phenotypes of disrupted* Shh* signaling [44]. Interestingly, many phenotypes of this mutant resembled those of a compound mutant that we reported to have a disrupted gradient of* Shh* signaling [7]. These results suggest that cilia play important roles to produce a specific gradient of* Shh* signaling.

In avian species, there are several well-studied naturally occurring mutants named* talpids* (*talpid*,* talpid* ^*2*^, and* talpid* ^*3*^), which exhibit various developmental defects, including craniofacial abnormalities [45, 46]. In particular,* talpid* ^*2*^ shows bilateral clefting between the frontonasal process and LNP, and a causative mutation has been identified in the* C2CD3* gene, which is important for ciliogenesis [46]. These results indicate that* Shh* signaling, which is mediated by cilia, is essential for lip formation. However, it is currently unknown how different cilial proteins can affect craniofacial development. In addition, there are different signaling pathways which are known to be regulated by primary cilia [47], and further comprehensive analyses are necessary to link the cilial defects to craniofacial disorders like CL.

## 6. Critical Interaction between* Shh* and* Wnt* Signaling During Lip Fusion

Canonical* Wnt* signaling is indispensable for normal facial development, including lip fusion. In both humans and mice, disruption of* Wnt* signaling has been shown to cause CL/P [48–50]. Additionally, the* P63*-*Irf6* signaling pathway has been identified to be activated by canonical* Wnt* signaling, which was associated with the growth and fusion of facial processes growth and fusion [51]. In our recent study, we discovered that* Shh* signaling (*Ptch1-LacZ*) and canonical* Wnt* signaling (*Topgal*) showed a complementary expression pattern during craniofacial development (Figures 2(c), 2(d), and 2(g)). Furthermore, we proved that enhanced* Shh* signaling could result in CL by inhibiting canonical* Wnt* signaling [7]. Previous research showed that genetic elimination of* Kif3a* from cranial neural crest cells in mice led to enhanced* Shh* signaling, together with disturbed canonical* Wnt* signaling in a tissue- and time-dependent manner [41]. These results strongly suggest that there is a critical interaction between* Shh* and canonical* Wnt* signaling during craniofacial development, and this is indispensable for proper lip formation.

## 7. Ethanol Exposure and* Shh* Signaling Affect Lip Formation

The etiology of CL is known to include both genetic and environmental factors [52]. One of the most well-studied maternal environmental factors that can lead to craniofacial abnormalities is ethanol exposure. Ethanol exposure of the developing embryo is known to cause craniofacial and brain deformities which resemble the phenotype of disrupted* Shh* signaling [53]. There are reports showing that embryonic ethanol exposure disrupts* Shh* signaling and enhances cell death in the prechordal mesendoderm and cranial neural crest cells [28, 54]. Recent studies showed the interaction between* Shh* signaling and ethanol exposure by demonstrating a significantly enhanced phenotype in* Shh* or* Gli2* heterozygous knockout mice that were treated with ethanol [55]. There was another report that showed that disruption of* Cdon*, one of the receptors for* Shh* signaling, together with ethanol exposure, would result into holoprosencephaly, while* Cdon* knockout mice did not exhibit this phenotype without the environmental insult [56]. This synergistic effect with* Cdon* could be restored by removing one copy of* Patched1* [57]. These results clearly demonstrate that there is a molecular and environmental interaction between* Shh* signaling and ethanol exposure during craniofacial development.

## Figures and Tables

**Table 1:** Mouse mutants that were used for investigating *Shh *signaling and lip development.

Mutated gene	Type of mutation	Phenotype	Reference
*Patched1 *	ENU	CL	[7]
*Patched1*	CKO (neural crest)	CL	[33]
*Smo *	CKO (neural crest)	Truncated face	[35]
*Shh *	Ectoderm overexpression	Malformed lip	[36]
*Boc, Gas1*	Double KO	CL/P	[31]
*Kif3a*	CKO (neural crest)	Hypertelorism, CP	[41]
*Ift172*	ENU	CP	[42]
*Ift144*	ENU	CL/P	[44]

ENU: *N*-ethyl-*N*-nitrosourea. CKO: conditional knockout. KO: knockout. CL: cleft lip. CP: cleft palate. CL/P: cleft lip and palate.
